# Biochemical and Computational Insights on a Novel Acid-Resistant and Thermal-Stable Glucose 1-Dehydrogenase

**DOI:** 10.3390/ijms18061198

**Published:** 2017-06-05

**Authors:** Haitao Ding, Fen Gao, Yong Yu, Bo Chen

**Affiliations:** 1 Key Laboratory for Polar Science of State Oceanic Administration, Polar Research Institute of China, Shanghai 200136, China; yuyong@pric.org.cn (Y.Y.); chenbo@pric.org.cn (B.C.); 2 East China Sea Fisheries Research Institute, Shanghai 200090, China; gaofen2011@163.com

**Keywords:** *Bacillus*, glucose 1-dehydrogenase, acid-resistant, thermal-stable, molecular dynamics simulation

## Abstract

Due to the dual cofactor specificity, glucose 1-dehydrogenase (GDH) has been considered as a promising alternative for coenzyme regeneration in biocatalysis. To mine for potential GDHs for practical applications, several genes encoding for GDH had been heterogeneously expressed in *Escherichia coli* BL21 (DE3) for primary screening. Of all the candidates, GDH from *Bacillus* sp. ZJ (BzGDH) was one of the most robust enzymes. BzGDH was then purified to homogeneity by immobilized metal affinity chromatography and characterized biochemically. It displayed maximum activity at 45 °C and pH 9.0, and was stable at temperatures below 50 °C. BzGDH also exhibited a broad pH stability, especially in the acidic region, which could maintain around 80% of its initial activity at the pH range of 4.0–8.5 after incubating for 1 hour. Molecular dynamics simulation was conducted for better understanding the stability feature of BzGDH against the structural context. The in-silico simulation shows that BzGDH is stable and can maintain its overall structure against heat during the simulation at 323 K, which is consistent with the biochemical studies. In brief, the robust stability of BzGDH made it an attractive participant for cofactor regeneration on practical applications, especially for the catalysis implemented in acidic pH and high temperature.

## 1. Introduction

NAD(P)-dependent glucose 1-dehydrogenase (GDH, EC 1.1.1.47) is an oxidoreductase present in various organisms and involved in glucose metabolic pathways, catalyzing the oxidation of d-glucose to d-glucono-1,5-lactone while simultaneously reducing NAD(P) to NAD(P)H [1,2,3,4,5,6]. As a member of the short-chain dehydrogenases/reductases family (SDRs), GDH is a tetrameric protein consisting of four identical subunits, which shares similar overall folding and oligomeric architecture with those of its homologous counterparts [7,8]. Due to the dual cofactor specificity, high activity, easy preparation, and cheap substrate, GDH has been widely used in biocatalysis [9,10,11], bioremediation [12], biosensors [13], and biofuel cells [14].

Biocatalysis has been considered as a powerful tool for the pharmaceutical and fine chemical synthetic processes due to the chemo-, regio-, and stereo-selectivity of enzymes [15]. However, because many kinds of industrial enzymes are cofactor-dependent, the enzymatic synthesis is limited by the considerable expenses of the cofactors. To tackle the issue of manufacturing expense on biocatalysis, several cofactor regeneration approaches have been proposed, of which the enzymatic regeneration method has been considered as an effective technique [16]. Due to the activity toward both NAD and NADP, GDH has been proposed as a promising candidate for coenzyme regeneration [17,18], compared with other oxidoreductases such as formate dehydrogenase [19], alcohol dehydrogenase [20], glucose-6-phosphate dehydrogenase [21], and phosphite dehydrogenase [22]. 

Although GDHs from various microorganisms have been employed as coenzyme regenerators for biocatalysis [9,10,11], new enzymes with robust stability against broad temperature and pH range are still preferred. In this work, a novel NAD(P)-dependent glucose 1-dehydrogenase from *Bacillus* sp. ZJ (BzGDH), with considerable acidic tolerance and thermal stability, has been extensively characterized through biochemical experiments. In contrast to previously reported acid-resistant GDHs, including GDH from *Bacillus thuringiensis* M15 (BtGDH) [3], *Bacillus* sp. G3 (BgGDH) [23], and *Bacillus cereus* var. *mycoides* (BcGDH) [24], BzGDH exhibited superior thermal stability to its homologous counterparts. To better understand this remarkable feature that distinguishes BzGDH from other acid-resistant GDHs, molecular dynamic (MD) simulation was conducted to investigate the conformational flexibility and fluctuations of BzGDH over time and spatial scales. Analysis of the trajectory shows that BzGDH is stable and can maintain its overall structure against heat during the simulation at 323 Kelvin (K), which is in accordance with the biochemical studies.

## 2. Results and Discussion

### 2.1. Sequence Analysis

**Figure 1:**
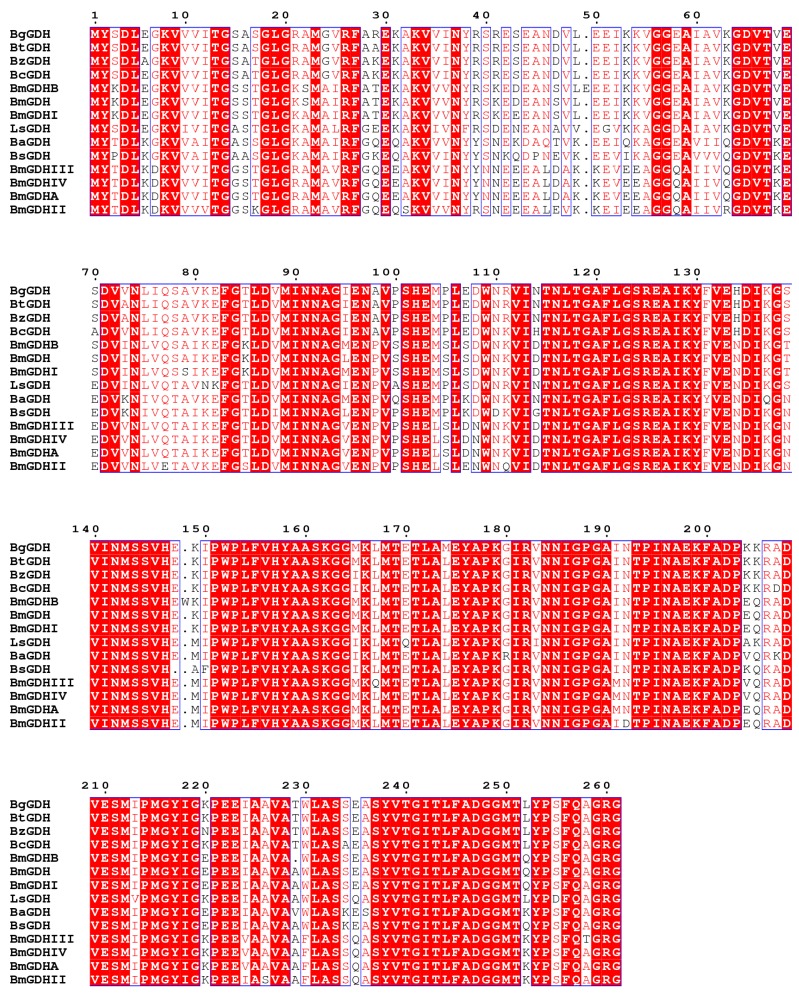
Multiple alignment of the primary structure of glucose 1-dehydrogenases (GDHs). Identical residues and conserved substitutions are shaded red and enveloped by rectangles, respectively. GDHs from *Bacillus* sp. ZJ (this study), *Bacillus megaterium* IWG3 [5], *Lysinibacillus sphaericus* G10 [2], *Bacillus cereus* var. *mycoides* [24], *Bacillus* sp. G3 [23], *Bacillus amyloliquefaciens* SB5 [1], *Bacillus thuringiensis* M15 [26], and *Bacillus subtilis* W168 [27] are abbreviated as BzGDH, BmGDH, LsGDH, BcGDH, BgGDH, BaGDH, BtGDH, and BsGDH, respectively. BmGDHA and BmGDHB are from *Bacillus megaterium* M1286 [6]. BmGDHI, BmGDHII, BmGDHIII, and BmGDHIV are from *Bacillus megaterium* IAM1030 [4,5]. Alignment of multiple protein sequences was conducted by using the Clustal X 2.0 program [28] and rendered by ESPript [29].

**Figure 2:**
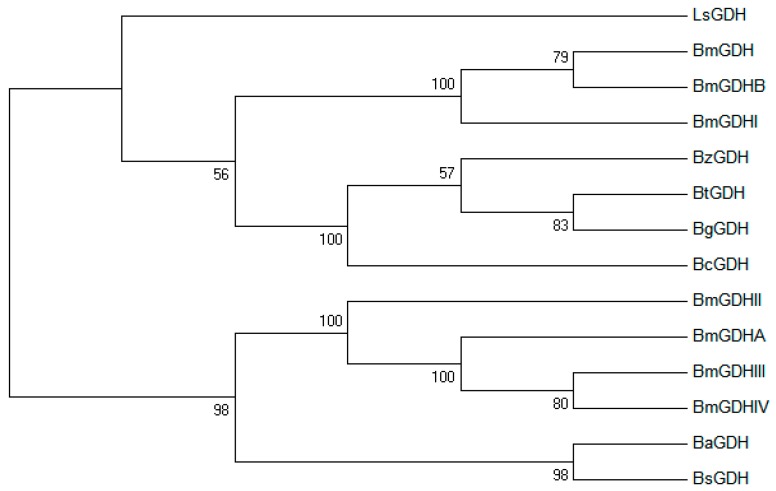
Unrooted phylogenetic tree of GDHs. The phylogenetic tree was constructed using the neighbor joining method [30] in MEGA7 software [31], with a bootstrap test of 1000 replicates. The evolutionary distances were computed using the Poisson correction method [32] and are in the units of the number of amino acid substitutions per site.

The gene *bzgdh* encodes a peptide consisting of 261 amino acids with a predicted molecular weight of 28 kDa and a theoretical isoelectric point of 5.4. Significant Pfam-A matches [25] revealed that BzGDH was affiliated to adh_short_C2 family (PF13561, Enoyl-(Acyl carrier protein) reductase), which belonged to the FAD/NAD(P)-binding Rossmann fold superfamily (CL0063), as well as other GDHs. BzGDH also shared the conserved coenzyme-binding GXXXGXG motif (14–20) and catalytic triad (Ser145/Tyr158/Lys162) with other GDHs. In addition, amino acid substitutions mostly occurred at the N-terminus of GDHs (Figure 1), indicating that the N-terminal sequence is less conservative than the C-terminal sequence, which played critical roles in substrate recognition. Phylogenetic analysis showed that these GDHs diverged into two clusters, and BzGDH belonged to the sub-branch consisting of BtGDH, BcGDH, and BgGDH (Figure 2), of which all exhibited acidic resistance in previous studies, suggesting that these four GDHs might originate from the same ancestral sequences.

### 2.2. Heterologous Expression and Purification

**Figure 3:**
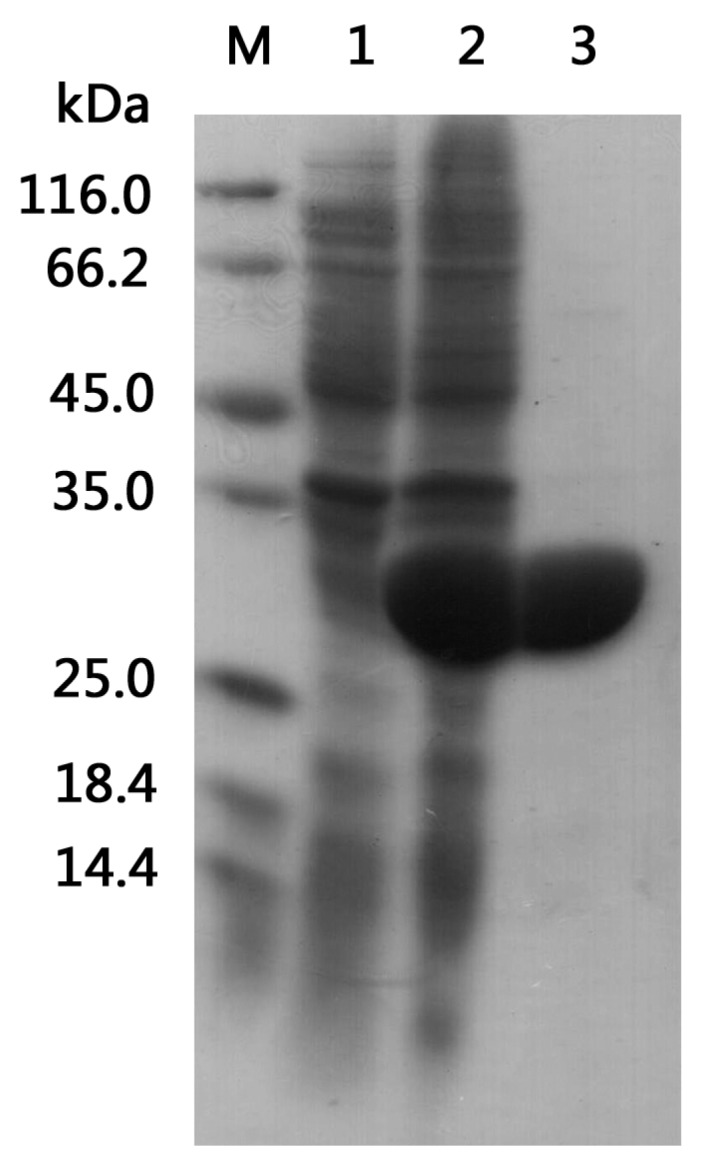
SDS-PAGE (sodium dodecyl sulfate polyacrylamide gel electrophoresis) analysis of total cell lysate and the purified enzyme. Lane M: protein molecular weight marker. Lane 1: uninduced total cell lysate of BzGDH. Lane 2: induced total cell lysate of BzGDH. Lane 3: purified BzGDH.

The specific activity of the purified BzGDH was 194 ± 2 U·mg^−1^ at 25 °C using NAD (nicotinamide adenine dinucleotide) as a cofactor. SDS-PAGE (sodium dodecyl sulfate polyacrylamide gel electrophoresis) analysis showed a homogeneous band corresponding to 30 kDa (Figure 3). By using gel filtration chromatography through a Zorbax Bio-series GF-450 column, the molecular weight of the native BzGDH was estimated to be 120 kDa. These results indicated that BzGDH was a homo-tetramer composed of four identical subunits, as well as other NAD-dependent GDHs derived from *Bacillus* [1,2,3,4,5,6,23,24,26,27].

### 2.3. Effects of pH and Temperature on the Activity and Stability

**Figure 4:**
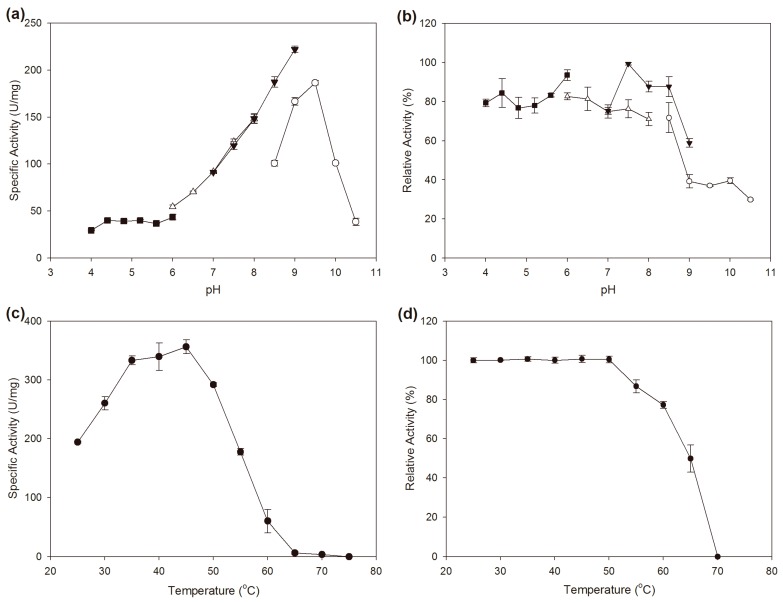
Effects of pH and temperature on the activity and stability of BzGDH. (**a**) Effect of pH on the activity of BzGDH; (**b**) Effect of pH on the stability of BzGDH. (■) pH 4.0–6.0, 100 mM sodium citrate buffer; (Δ) pH 6.0–8.0, 100 mM sodium phosphate buffer; (▼) pH 7.0–9.0, 100 mM Tris-HCl buffer; (○) pH 8.5–10.5, 100 mM glycine-NaOH buffer; (**c**) Effect of temperature on the activity of BzGDH; (**d**) Effect of temperature on the stability of BzGDH.

BzGDH exhibited activity at a wide pH range from 4.0 to 10.5, and displayed maximum activity at pH 9.0 in Tris-HCl buffer among all buffers. Actually, the chemical composition of the sodium citrate, sodium phosphate, and Tris-HCl buffers, showed no significant influence on the specific activity of the enzyme, and the differences in the specific activity are mainly caused by the change of the pH of the solution. However, a significant decrease of specific activity was observed in the glycine-NaOH buffer at pH values of 8.5 and 9.0 when compared to those of the same pH values of the Tris-HCl buffer, indicating that glycine might inhibit the activity of BzGDH. Surprisingly, the optimum pH was determined as 9.5 in Glycine-NaOH buffer (Figure 4a), which is inconsistent with the maximum activity pH of 9.0. A reasonable explanation for this discrepancy is that the observed activity of the enzyme is not only affected by the p*K*a of its catalytic residues which played critical roles on the activity, but is influenced by the stability of the enzyme which might be unstable at its optimum pH (Figure 4b), and is even sometimes affected by the chemicals in the buffer such as glycine in this case. In regards to its pH stability, BzGDH was stable over a broad pH range, especially in the acidic region, which could maintain around 80% of its initial activity in the pH range of 4.0–8.5 after incubating for 1 hour (Figure 4b).

As demonstrated in Figure 4c, the optimum catalytic temperature of BzGDH was determined as 45 °C. The activity of BzGDH decreased linearly from 45 to 65 °C and could not be measurable at 75 °C. In consistent with its higher optimum reaction temperature, the recombinant enzyme also possessed good thermal stability, which was stable after incubation at temperatures below 50 °C for 30 min and still maintained 50% of its initial activity after incubation at 65 °C for 30 min (Figure 4d). BzGDH exhibited superior thermal stability to its homologous counterparts, BgGDH [23] and BcGDH [24], which were almost completely inactivated after incubation at 50 °C without any protective agent.

Since stability is an indispensable characteristic for the utilization of enzymes in real life, the considerable stability of BzGDH against both heat and acid made it a very promising candidate in practical application in harsh conditions.

### 2.4. Substrate Specificity and Steady-State Kinetics

As shown in Table 1, the substrate spectrum of BzGDH was similar to that of BcGDH. However, both BzGDH and BcGDH displayed stricter substrate specificity toward various sugars than that of BgGDH, especially for galactose and mannose, indicating that BzGDH could be a potential diagnostic reagent for blood glucose measurement as well as BcGDH.

The steady-state kinetic constants of BzGDH were determined by using a nonlinear fitting plot (Table 2). Although BzGDH had similar *k_cat_* values for both NAD and NADP, the *K_m_* value for NADP was 5.6-fold higher than that for NAD, indicating that BzGDH preferred NAD rather than NADP as the cofactor. The cofactor preference of BzGDH resembled that of BmGDHIII, BmGDHIV [4], and BtGDH [3], while BmGDH, BmGDHI, BmGDHII [5], and BgGDH [23] preferred NADP.

### 2.5. Homology Modeling and Electrostatic Potential Analysis

**Figure 5:**
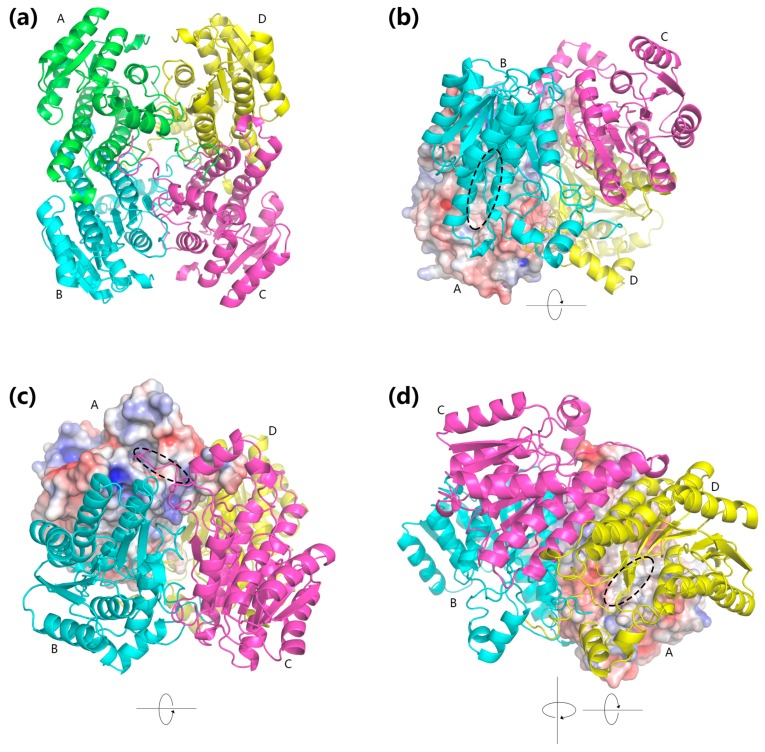
±5 kT/e electrostatic potential surface of BzGDH. (**a**) Tetrameric structure of BzGDH; (**b**) Electrostatic potential surface representation of the interface between subunits A and B; (**c**) Electrostatic potential surface representation of the interface between subunits A and C; (**d**) Electrostatic potential surface representation of the interface between subunits A and D. Subunits ABCD were labeled using the corresponding capital letters nearby, respectively. Positive, negative, and neutral electrostatic potential surfaces are rendered by blue, red, and white, respectively. The non-polar regions of the contact surfaces of subunits AB, AC, and AD were circled by dashed ellipses.

The quaternary structure of BzGDH was constructed by SWISS-MODEL [33] and evaluated by ProSA-web [34] and PROCHECK [35]. Both of the Z-score and Ramachandran plot statistics indicated that the dimensional structure of BzGDH (Figure 5a) had been modeled reasonably (Table 3). To investigate the electrostatic potential of BzGDH, the model of BzGDH was subjected to the software APBS [36] and PyMOL (The PyMOL Molecular Graphics System, Version 1.7 Schrödinger, LLC. available online: http://pymol.org/), to generate the electrostatic potential molecular surface. As shown in Figure 5, the contact surfaces of subunits AB, AC, and AD circled by black ellipses are mainly constituted by non-polar amino acid residues and are surrounded by acidic amino acid residues. The non-polar areas can maintain their electrically neutral state in either acidic or alkaline solutions, whereas the acidic areas would be negatively charged in alkaline solutions, leading to the mutual repulsion between subunits. Therefore, the acid-resistance of BzGDH could be explained by the electrostatic potential of contact surfaces between subunits, as well as BcGDH [24].

### 2.6. Global Structure Stability

**Figure 6:**
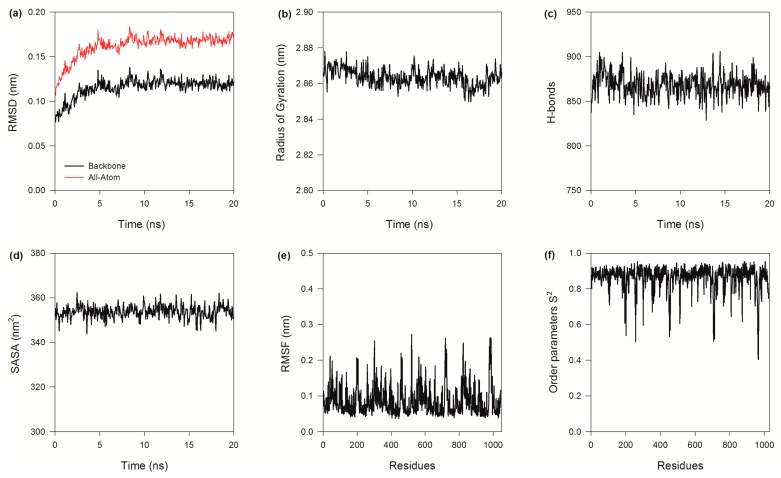
Dynamic changes of BzGDH in the molecular dynamics (MD) simulation. (**a**) All-atom and backbone-atom root mean square deviation (RMSD) as functions of time; (**b**) Radius of gyration as a function of time; (**c**) Hydrogen bonds as a function of time. Hydrogen bonds were detected by GROMACS (GROningen MAchine for Chemical Simulations) with default geometrical criterion, which defined both the donor-acceptor distance (≤0.35 nm) and the hydrogen-donor-acceptor angle (≤30 °C); (**d**) Solvent accessible surface areas (SASA) as a function of time; (**e**) Root mean square fluctuation (RMSF) as a function of residue numbers. (**f**) N-H generalized order parameter *S*^2^ as a function of residue numbers.

To study the stability and mobility of BzGDH, the model was subjected to a 20-ns MD simulation at 323 K. The stability of BzGDH was analyzed by the all-atom and backbone-atom root mean square deviation (RMSD), respectively, both of which increased from the beginning of the simulation and reached an equilibrium state at about 10 ns (Figure 6a), suggesting no significant structural changes for BzGDH during the simulation. In addition, the radius of gyration, the hydrogen bonds of intra-protein, and the solvent accessible surface area (SASA) of BzGDH all displayed steadily dynamic changes against time (Figure 6b–d), further confirming the stable global behavior of BzGDH during the simulation at 323 K.

### 2.7. Structure Flexibility 

**Figure 7:**
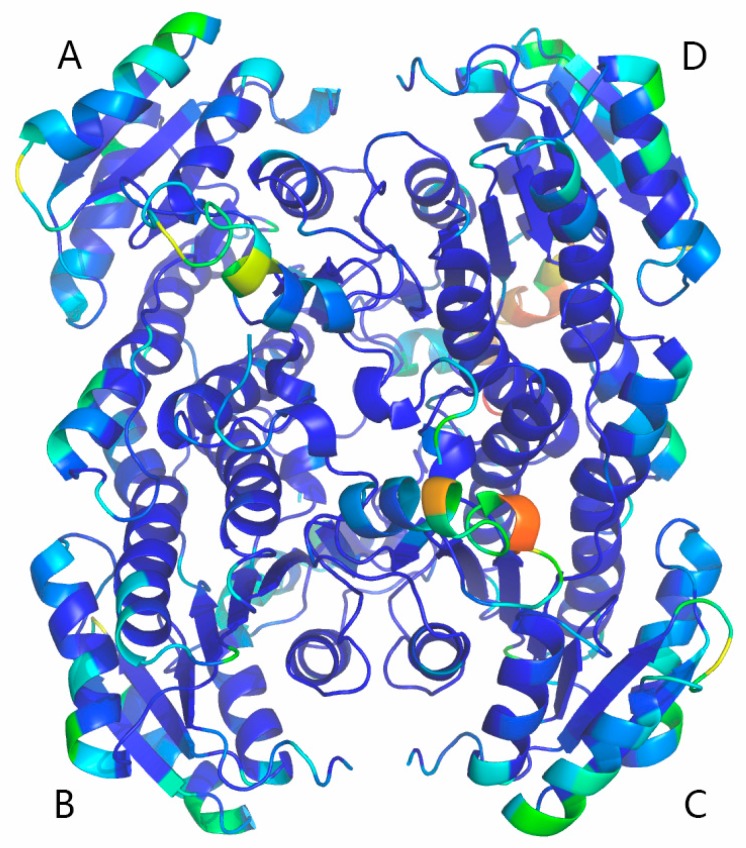
Cartoon representation of BzGDH shaded according to the B-factors (temperature factor) of each residue. Subunits ABCD were labeled using the corresponding capital letters nearby. The structure was shaded from the blue to red spectrum along with the increase of B-factor values from 3.98 to 193.74.

The conformational flexibility of BzGDH was assessed using the root mean square fluctuation (RMSF) of C-alpha (Cα) atoms per residue. Generally, regular secondary structure regions display tiny fluctuations with small RMSF values during the simulation, whereas prominent fluctuations with large RMSF are observed for irregular secondary structure regions such as terminal or loop regions, which often bear certain function of proteins. As shown in Figure 6e, regions involved in coenzyme binding (39–55) and substrate binding (190–210) of each subunit are more flexible with large RMSF values than other regions. The RMSF values were converted to B-factors using the equation:

B-factor = (8 × π^2^ × RMSF^2^)/3 (1)

to visualize global structure rigidity and flexibility of BzGDH. As shown in Figure 7, most regions of BzGDH are rigid, except for the aforementioned flexible regions, indicating that the enzyme is stable during the simulation at 323 K.

In addition to the observation of RMSF, the bond-specific fluctuations in protein structure can further be captured by the Lipari–Szabo order parameter S^2^ [37], which provide an intuitive description of the amplitude of spatial restriction of the internal motions of the bond vectors on a fast timescale from picosecond to nanosecond (ps-ns). More specifically, S^2^ represents the component of the H-X bond vector autocorrelation function which is dissipated by global molecular tumbling, while (1 − S^2^) characterizes the bond vector orientational disorder arising from internal motion occurring more rapidly than the molecular tumbling. The S^2^ order parameter can range from 0 to 1, with 1 corresponding to a rigid bond vector (completely restricted) and 0 corresponding to the highest degree of disorder for a bond vector (completely isotropic). Higher order parameters (0.85) were observed in the regions of secondary structure, while unstructured regions showed lower order parameters (0.4–0.6).

The order parameter S^2^ of the main chain N-H bonds of BzGDH has been calculated based on the equilibrium MD trajectories. The average value of the order parameter S^2^, over all residues, is 0.86 for BzGDH. The most flexible region that showed lower S^2^ of each subunit is the substrate binding domain, with residues Lys 179, Gly 180, Arg 182, Asn 184, Asn 185, Ala 190, Asn 196, and Asp 202 involved, indicating that these residues exhibit considerable disorder on the ps-ns timescale. Similarly, residues Gln 257, Ala 258, and Gly 259 in the C-terminal region of the protein have low order parameters, also implying that this region is disordered on the ps-ns timescale. Indeed, the order parameter revealed that these regions are flexible on the ps–ns timescale, with the fluctuations functioning to allow substrate access to and release of products from the active site. The results of the computation of the order parameters are in considerable agreement with the RMSF profiles, with the greatest flexibility occurring in loop regions, while other secondary structural elements are more constrained.

### 2.8. Essential Dynamics

**Figure 8:**
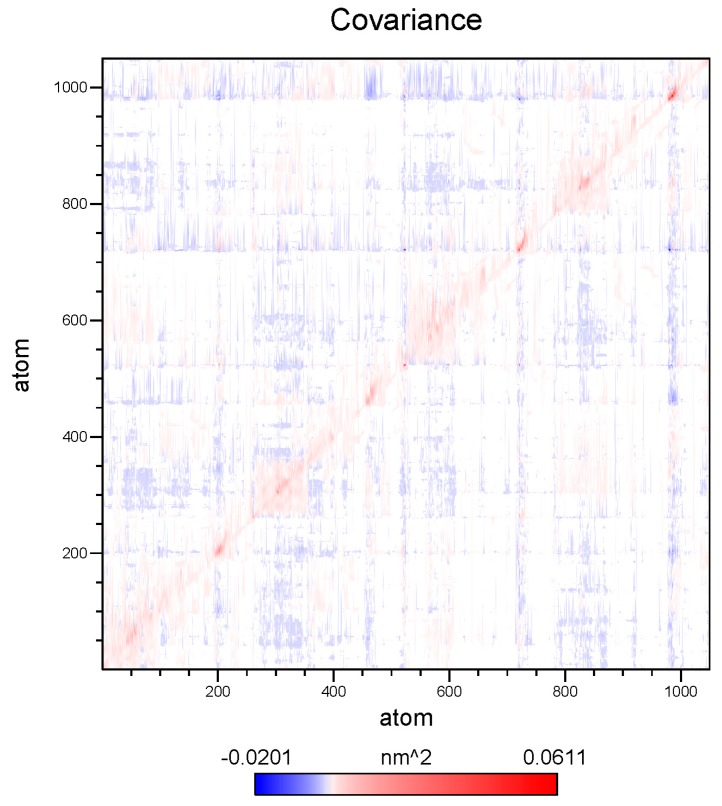
Covariance analysis of the atomic fluctuation of BzGDH in the MD (molecular dynamics) simulation. The correlation matrix is computed using the C-α Cartesian coordinates. The collective motions between pairs of residues are represented as red for correlated, white for uncorrelated, and blue for anti-correlated motions, respectively. The amplitude of fluctuation was represented by the color depth.

To reveal the concerted fluctuations of BzGDH over time and spatial scales, essential dynamics (ED) is employed to extract information from sampled conformations over the molecular dynamics trajectory [38]. Practically, the essential dynamics of a protein is obtained by performing principal component analysis (PCA), which is a multivariate statistical technique involving diagonalization of the covariance matrix (Figure 8) constructed from atomic displacements of Cα atoms, to reduce the number of dimensions required to describe protein dynamics and yield a set of eigenvectors that provide information about collective motions of the protein [39].

**Figure 9:**
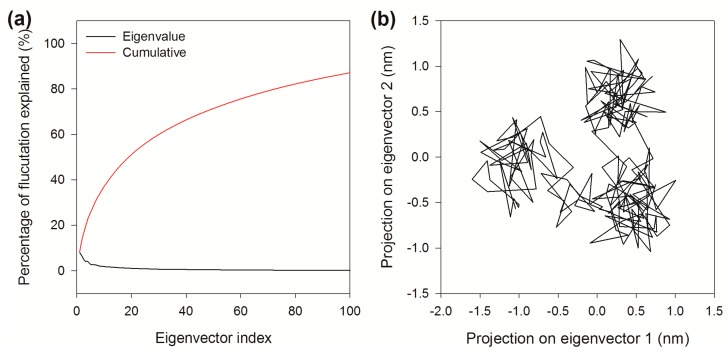
Principal component analysis of BzGDH in the MD simulation. (**a**) Relative cumulative deviation up to the first 100 eigenvectors provided by the essential dynamics analysis performed on the Cα atoms of BzGDH; (**b**) Projections of the trajectory on the plane defined by the first two principal eigenvectors. Horizontal axis: atomic displacement along the first eigenvector. Vertical axis: atomic displacement along the second eigenvector.

The eigenvectors represent the directions of motion, and the corresponding eigenvalues represent the amount of motion along each eigenvector, where larger eigenvalues describe motions on larger spatial scales. Generally, the first 10–20 eigenvectors are enough to capture the principal motions of the protein and describe more than 90% of all cumulative protein fluctuations [40]. However, it can be seen that only 14.3% of the total Cα motion can be explained by the first two eigenvectors, even the first 20 eigenvectors merely contribute for 51.2% of the total Cα motion from Figure 9a. This shows that most of the internal motions of BzGDH are not confined within a subspace of small dimension, and no obvious collective motion of the backbone of BzGDH is observed from the MD simulation performed at 323 K, reflecting that the enzyme can maintain its overall structure against heat, which is in accordance with the biochemical experiments.

**Figure 10:**
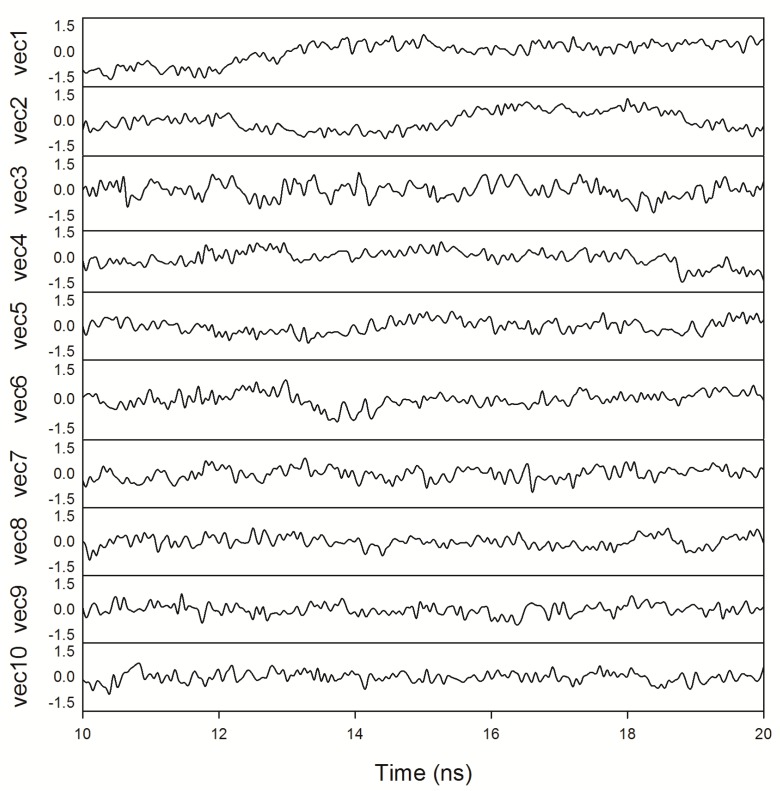
Motions along the first ten eigenvectors obtained from the Cα coordinates’ covariance matrix.

Figure 9b shows the trajectory projected on the plane defined by the first two principal eigenvectors. The trajectories filled most of the expected ranges, suggesting the deficiency of a coupled force field, which leads to independent motions. The trajectories were projected onto the individual eigenvectors against time to further investigate the motion along the eigenvector directions. It is clear from Figure 10 that the fluctuations of the first six eigenvectors are relatively large, whereas those of the subsequent eigenvectors become successively flat, indicating that the motions belong to the last four eigenvectors have reached their equilibrium fluctuation, which cannot be used to describe the motions of the system. Due to the limitation of hardware, such simulations may not capture the essential motions related to function at much longer timescales. Improvements in computational power will fill the gap between reality and simulation.

## 3. Materials and Methods

### 3.1. Strains, Plasmids, and Chemicals

Strain *Bacillus* sp. ZJ isolated from the soil near Yuhangtang River in Hangzhou, China, was used as a source for retrieving glucose 1-dehydrogenase. *Escherichia coli* (*E. coli*) DH5α and BL21 (DE3), expression vector pET28a (+), and Ni-NTA resin were purchased from Invitrogen. Taq DNA polymerase, PrimeSTAR HS DNA Polymerase, T4 DNA ligase, *Nde*I, and *BamH*I were purchased from TaKaRa. Genomic DNA, plasmid and gel extraction kits were purchased from Axygen. All other chemicals were of analytical grade.

### 3.2. Cloning of the Bzgdh Gene and Sequence Analysis

The gene *bzgdh* was amplified by using genomic DNA of *Bacillus* sp. ZJ as a template with the forward primer 5′-GGAATTCCATATGTATAGTGATTTAGCAGG-3′ and the reverse primer 5′-CGGGATCCTATTACCCACGCCCAGC-3′, which carried cutting sites of *Nde*I and *BamH*I (underlined), respectively. The amplified fragments were digested with *Nde*I and *BamH*I simultaneously, then purified by using a gel extraction kit prior to ligate with the pre-digested vector pET-28a (+). The recombinant plasmid harboring gene *bzgdh* was transformed into competent cells of *E. coli* DH5α for sequencing.

Homologous searches in GenBank were performed using the BLAST server (available online: http://blast.ncbi.nlm.nih.gov). Alignment of multiple protein sequences was conducted by using the Clustal X 2.0 program [28] and rendered by ESPript [29]. The phylogenetic tree was constructed using the neighbor-joining method in MEGA7 [31], with a bootstrap test of 1000 replicates.

The nucleotide sequence for GDH of *Bacillus* sp. ZJ was deposited in GenBank under accession number KJ701281.

### 3.3. Expression and Purification of Recombinant BzGDH

The recombinant plasmid was transformed into competent cells of *E. coli* BL21 (DE3) for expression. The recombinant cells were cultivated in Luria-Bertani broth containing 50 µg kanamycin·mL^−1^ at 37 °C with a shaking speed of 250 rpm. The expression of recombinant protein was induced by adding 0.5 mM of IPTG to the medium when the OD_600_ of the culture reached 0.5–0.8, followed by another 12 h incubation at 25 °C with a shaking speed of 200 rpm. The cells were harvested by centrifugation at 10,000× *g* for 10 min at 4 °C and were washed with the binding buffer (50 mM NaH_2_PO_4_, 500 mM NaCl, 20 mM imidazole, pH 8.0), and then lysed by ultrasonication. The cell debris was removed by centrifugation at 15,000× *g* for 30 min at 4 °C, and then the supernatant was loaded onto a column containing pre-equilibrated Ni-NTA resin. The column was washed with binding buffer and subsequently eluted with elution buffer (50 mM NaH_2_PO_4_, 500 mM NaCl, 250 mM imidazole, pH 8.0). The eluted enzyme was desalted and concentrated by ultrafiltration and stored at −80 °C in 25 mM sodium phosphate buffer (pH 6.5) with 30% of glycerol contained. The protein concentration was determined by Bradford’s method using bovine serum albumin as the reference.

Denaturing discontinuous polyacrylamide gel electrophoresis was performed on a 5% stacking gel and a 12% separating gel. The native molecular weight of GDH was determined by size-exclusion chromatography according to the protocol of the manufacture (Zorbax Bio-series GF-450, Agilent, Santa Clara, CA, USA), using lysozyme (14.3 kDa), chicken ovalbumin (45 kDa), bovine serum albumin fraction V (67 kDa), and goat IgG (150 kDa) as standards.

### 3.4. Enzyme Activity Assay

Glucose dehydrogenase activity was determined by assaying the absorbance of NADH at 340 nm in 100 mM sodium phosphate (pH 8.0) containing 200 mM glucose and 1 mM NAD at 25 °C. All measurements were conducted in triplicate. One unit of enzyme activity was defined as the amount of the enzyme that catalyzed the formation of 1 μmol of NADH per minute.

### 3.5. Effects of pH and Temperature on the Activity and Stability of BzGDH

The optimum pH of BzGDH was measured at pH ranging from 4.0 to 10.5 at 25 °C. The effect of pH on the stability of BzGDH was determined by measuring the residual activity after incubating BzGDH in buffers with different pH values for one hour at 25 °C.

The optimal temperature of BzGDH was determined at different temperatures (25–75 °C) in phosphate buffer at pH 7.0. The thermal stability of BzGDH was assayed by measuring the residual activity after incubating BzGDH at different temperatures (25–75 °C) in phosphate buffer at pH 7.0 for 30 min.

### 3.6. Substrate Specificity of BzGDH

The substrate specificity of BzGDH was determined by the aforementioned enzyme activity assay, except that glucose was replaced by sucrose, lactose, maltose, xylose, galactose, mannose, fructose, and arabinose, respectively.

### 3.7. Steady-State Kinetics of BzGDH

In order to obtain the kinetic constants for the coenzyme, 200 µM of glucose was employed as the substrate and 0.01 to 0.2 mM NAD and NADP were used as the coenzymes, respectively. For analysis of the kinetics for glucose, 1 mM NAD was used as a cofactor, 1 to 200 mM glucose was used as the substrate. GDH activity was measured as described above. The kinetic constants were determined by using a nonlinear fitting of the Michaelis-Menten equation:

*v* = (*V_max_* × [S])/(*K_m_* + [S]) (2)

where [S] is the concentration of the cofactor or substrate, *K_m_* is the Michaelis constants for the cofactor or substrate, *v* is the reaction velocity, and *V_max_* is the maximum reaction velocity. The turnover number *k_cat_* was calculated by the equation:

*V_max_* = *k_cat_* × [E] (3)

where [E] is the concentration of the enzyme. 

### 3.8. Homology Modeling and Electrostatic Potential of BzGDH

The crystal structure of glucose 1-dehydrogenase from *Bacillus megaterium* IWG3 (PDB code: 1GEE, 1.60 Å) [41], which shares 88.12% identity with BzGDH, was served as the template for homology modeling of BzGDH. The three-dimensional model of BzGDH was constructed by using the SWISS-MODEL [33]. Precise evaluation of the model quality was performed using ProSA-web [34] and PROCHECK [35]. The structure for electrostatics calculations was prepared by PDB2PQR [42] using the AMBER force field and assigned protonation states at pH 7.0. The electrostatic potential of BzGDH was calculated by APBS [36] using the linearized Poisson-Boltzmann equation (lpbe) at 298 K with the monovalent ion concentration of 0.1 M. The dielectric constants of protein and solvent were set as 2.0 and 78.0, respectively. The electrostatic potential molecular surface was represented by PyMOL. 

### 3.9. Molecular Dynamic Simulations of BzGDH

The constructed model of BzGDH was subjected to the software package GROMACS 5.0.2 [43], with the AMBER99SB [44] force field adopted, for molecular dynamics simulations. The model was first placed into the center of a virtual cubic box with a side length of 11.049 nm and solvated with 39,486 TIP3P water molecules. The pH condition was 7.0 according to the ionization state of the protein with a charge of −20, and twenty Na^+^ ions were added to the water box as counter ions to neutralize the negative charge of the entire system. Bond lengths were constrained by the LINCS algorithm to ensure covalent bonds to maintain their correct lengths during the simulation. Energy minimization of the system was conducted using the steepest descent algorithm for 5000 steps, followed by a 500-ps equilibration simulation with harmonic position restraints on the heavy protein atoms to equilibrate the solvent molecules around the protein. Subsequently, a 2-ns simulation without position restraints was conducted to equilibrate the entire system. Finally, the production simulation was performed for 20 ns at the target temperature. All simulations were performed under the NPT ensemble with periodic boundary conditions and a time step of 2 fs. The temperature of the system was kept at 323 K using the v-rescale method, and the pressure was kept at 1 bar using the Parrinello-Rahman method.

According to the RMSD profile of BzGDH, trajectories that reached the equilibrium state (10–20 ns) were used for further analysis. Principal component analysis was conducted to identify the direction and amplitude of the most prominent characteristics of the motions of BzGDH along the simulation trajectory. Generalized order parameters S^2^, employed as a measure of the degree of spatial restriction of motion, were also calculated for the N-H bonds of BzGDH.

## 4. Conclusions

In this study, a novel NAD(P)-dependent glucose 1-dehydrogenase from *Bacillus* sp. ZJ has been extensively characterized, with remarkable acidic tolerance and thermal stability. To better understand the stability feature of BzGDH against the structural context, molecular dynamics simulation was conducted to investigate the conformational flexibility and fluctuations of BzGDH over time and spatial scales. Analysis of the trajectory shows that BzGDH is stable and can maintain its overall structure against heat during the simulation at 323 K, which is in accordance with the biochemical studies. In brief, the robust stability of BzGDH made it a promising participant for cofactor regeneration in practical applications, especially for catalysis implemented in acidic pH and high temperature.

## Figures and Tables

**Table 1:** Substrate specificity of GDHs.

Substrate	Relative Activity (%) ^1^		
	BzGDH	BgGDH [23]	BcGDH [24]
d-glucose	100	100	100
d-galactose	6.8	22.0	7.3
d-mannose	3.2	7.1	4.4
d-fructose	0.9	0.6	0
d-xylose	6.1	6.4	6.0
d-arabinose	0	0.2	0
d-maltose	10.0	13.0	11.0
d-lactose	3.1	2.6	5.2
d-sucrose	0.9	6.3	2.51

^1^ The activities are expressed relative to those for d-glucose.

**Table 2:** Kinetic constants of BzGDH.

Substrate/Cofactor	*K_m_* (mM)	*k_cat_* (s^−1^) ^1^	*k_cat_*/*K_m_* (mM^−1^·s^−1^)
d-glucose	17.126 ± 0.946	87.844 ± 1.362	5.129
NAD	0.072 ± 0.009	84.521 ± 2.175	1166.294
NADP	0.404 ± 0.088	73.960 ± 2.677	182.978

^1^ The values of *k_cat_* were calculated for one subunit.

**Table 3:** Evaluation of models generated by homology modeling.

Model	Z-Score ^1^	Ramachandran Plot ^2^			
		Most Favored (%)	Additional Allowed (%)	Generously Allowed (%)	Disallowed (%)
1GEE	−8.87	91.2	8.0	0.9	0
BzGDH	−8.80	89.1	10.5	0.4	0

^1^ Calculated by ProSA-web [34]; ^2^ Calculated by PROCHECK [35].
